# Temperature Prediction Using Multivariate Time Series Deep Learning in the Lining of an Electric Arc Furnace for Ferronickel Production

**DOI:** 10.3390/s21206894

**Published:** 2021-10-18

**Authors:** Jersson X. Leon-Medina, Jaiber Camacho, Camilo Gutierrez-Osorio, Julián Esteban Salomón, Bernardo Rueda, Whilmar Vargas, Jorge Sofrony, Felipe Restrepo-Calle, Cesar Pedraza, Diego Tibaduiza

**Affiliations:** 1 Control, Modeling, Identification and Applications (CoDAlab), Department of Mathematics, Escola d’Enginyeria de Barcelona Est (EEBE), Campus Diagonal-Besòs (CDB), Universitat Politècnica de Catalunya (UPC), Eduard Maristany 16, 08019 Barcelona, Spain; 2 Departamento de Ingeniería Mecánica y Mecatrónica, Universidad Nacional de Colombia, Cra 45 No. 26-85, Bogotá 111321, Colombia; jsofronye@unal.edu.co; 3 Departamento de Ingeniería Eléctrica y Electrónica, Universidad Nacional de Colombia, Cra 45 No. 26-85, Bogotá 111321, Colombia; jfcamachoo@unal.edu.co (J.C.); dtibaduizab@unal.edu.co (D.T.); 4 Departamento de Ingeniería de Sistemas e Industrial, Universidad Nacional de Colombia, Cra 45 No. 26-85, Bogotá 111321, Colombia; cgutierrez@unal.edu.co (C.G.-O.); jesalomont@unal.edu.co (J.E.S.); ferestrepoca@unal.edu.co (F.R.-C.); capedrazab@unal.edu.co (C.P.); 5 South32-Cerro Matoso S.A., Km 22 Highway SO Montelibano, Córdoba 234001, Colombia; Bernardo.S.Rueda@south32.net (B.R.); whilmar.p.vargas@south32.net (W.V.)

**Keywords:** temperature prediction, electric arc furnace, deep learning, structural health monitoring, gated recurrent unit, GRU, multivariate time series

## Abstract

The analysis of data from sensors in structures subjected to extreme conditions such as the ones used in smelting processes is a great decision tool that allows knowing the behavior of the structure under different operational conditions. In this industry, the furnaces and the different elements are fully instrumented, including sensors to measure variables such as temperature, pressure, level, flow, power, electrode positions, among others. From the point of view of engineering and data analytics, this quantity of data presents an opportunity to understand the operation of the system under normal conditions or to explore new ways of operation by using information from models provided by using deep learning approaches. Although some approaches have been developed with application to this industry, it is still an open research area. As a contribution, this paper presents an applied deep learning temperature prediction model for a 75 MW electric arc furnace, which is used for ferronickel production. In general, the methodology proposed considers two steps: first, a data cleaning process to increase the quality of the data, eliminating both redundant information as well as atypical and unusual data, and second, a multivariate time series deep learning model to predict the temperatures in the furnace lining. The developed deep learning model is a sequential one based on GRU (gated recurrent unit) layer plus a dense layer. The GRU + Dense model achieved an average root mean square error (RMSE) of 1.19 °C in the test set of 16 different thermocouples radially distributed on the furnace.

## 1. Introduction

Structural health monitoring (SHM) remains a priority in large-scale industrial applications because of the multiple advantages in its implementation such as the use of sensors permanently attached to the structure for continuous monitoring and its readily available form for the application of data-driven approaches to determine the health of the structure under evaluation [1,2]. Structures in industrial processes require special attention since the inability to do so may put at risk its operation. Structural health uncertainty, in conjunction with variations in the operational or environmental conditions, increases the risk of accidents, which in turn may result in catastrophic events [3]. One example of structures subjected to extreme operational conditions is the furnaces used in the smelting industry where temperatures, pressures, flows, among other variables, can vary considerably due to changes in the inputs (e.g., chemical composition of the raw material) or the operational conditions. This implies that operators are obliged to continuously monitor the structural health of the furnace before applying any changes in the operation set points, in order to maintain conditions that allow the secure operation of the system and the health of the structure. SHM is of particular interest for electric arc furnaces (EAF), which are characterized by the action of heating the materials using a covered electric arc for the smelting process. Routine operation causes wear in the wall lining of the furnace, hence monitoring wall thickness is of special interest to avoid run-outs of the smelting material. One strategy to evaluate the state of the structure is to directly monitor the wall thickness via routine inspection using specialized techniques. However, it is an expensive and difficult task because of the scale of the system. For this reason, it is of interest to use other measurements that can indirectly account for the thickness of the wall lining. As it turns out, the measured temperature in the wall is a good indicator of its health and can be used for monitoring tasks in a continuous (on-line) way.

Some recent works that use machine learning techniques to predict temperature variables within the smelting process have addressed the problem from different points of view. Mishra et al. [4] compared five deep learning models for multivariate prediction of time series temperatures. The study yielded as a result that a deep convolutional network (DCN) performs best with wavelet and fast Fourier transform (FFT). An online estimation of electric arc furnace tap temperature by using fuzzy neural networks was developed by Fernandez et al. [5] and its application of this helped reduce the consumption of energy in an electric arc furnace. In the work of Fontes et al. [6], the hot metal temperature in a blast furnace was predicted using an approach based on fuzzy c-means (FCM) and exogenous nonlinear autoregressive model (NARX); the estimate was later implemented as a soft sensor for predicting temperature. In Shockaert and Hoyez [7] a multivariate time series approach was built with a deep generative CycleGAN model combined with a long short-term memory (LSTM)-based autoencoder (AE). Particularly, that approach handles a transfer learning methodology in which data obtained from a source furnace is used to train a model that can be used to evaluate data from a target furnace. The forecasting of the hot metal temperature in a blast furnace is shown in the work of Iffat et al. [8]. Specifically, an optimal time lag at which the input variables have an impact on the hot metal temperature is determined. Additionally, an incremental learning methodology that considers changes in raw material composition, process control methods, and aging equipment was developed.

In order to improve the efficiency in a blast furnace, a self-organizing Kohonen neural network approach was developed in [9]. This approach managed to complement the control of process operating parameters for the blast furnace process. An artificial neural network (ANN) model was applied to predict the slag and metal composition in a ferromanganese production unit with a submerged arc furnace [10]. The advantages of this ANN included the reduction of power and coke consumption. Similarly, in the work of Ducic et al. [11], an ANN was derived as an intelligent soft sensor in the process of white cast iron production. An increase in productivity, as well as material and energy efficiency, can be translated into a reduction of the environmental impact and cost of the steel-making process in a basic oxygen furnace (BOF); in [12], the aforementioned objectives were reached through the use of standard machine learning models to predict the end-point targets of variables like the final melt temperature and upper limits of the carbon and phosphorus content with minimum material loss. A random forest algorithm was used in [13] to improve the quality of steel casting for tire reinforcement; 140 process variables were used as features and the output can take values 0 or 1 depending on whether the casting was rejected or not. The best area under the receiver operating characteristics (AUROC) in the test set was 0.85 obtained by the random forest classification method. The prediction issue of the amount of alloying additives in order to obtain the desired chemical composition of white cast iron was solved applying a neural network model in [14]. A three-month-long monitoring of the metal melting process data set was used. Besides the data was split into training and test sets founding that the neural network model reached a mean squared error of 3.31% in the test set.

As previous works have shown, different variables can be predicted by using historical operation data to evaluate the health of furnaces, or to predict the behavior of the process when inputs are changed. Therefore, the development of prediction models becomes one of the main necessities in the areas of operation analysis, control, and maintenance of EAFs and remains an open research area. As a contribution, this work presents the development of a deep learning model to predict the temperature in the lining of the EAF. Details about the experimental setup, the data acquisition from sensors, its preprocessing step, model development, and its validation are also presented in this work. This work was carried out in a joint effort between academia and industry, Universidad Nacional de Colombia and Cerro Matoso S.A. (CMSA). The interested reader can acquire some more background of the research process by reviewing some of the previous works developed by the authors, where the problems associated with sensor networks and continuous monitoring in this kind of furnace, including temperature monitoring [15,16], gap monitoring [17], and thickness monitoring [18] using ultrasonic and ground penetrating radar (GPR) methods are also tackled.

This paper is organized as follows: Section 2 includes a theoretical background where some concepts about the company, the process, and the methods used in the methodology are briefly introduced. Section 3 provides information about the developed methodology including a description of the dataset used for the validation. Section 4 presents the results and discussion; finally, conclusions are included in the last section.

## 2. Theoretical Background and Related Works

### 2.1. Cerro Matoso S.A.

As context to the work presented herein, Cerro Matoso S.A. (CMSA) is one of the world’s major producers of ferronickel and it is operated by South32. This is an open-cut mine operation in northern Colombia, with more than 35 years of operation in the region. More details about the process developed by CMSA can be found directly on its web page [19]. A brief description of nickel and the ferronickel metallurgical process are included next in order to contextualize the development of the methodology.

### 2.2. Nickel and Ferronickel Metallurgical Process Description

Nickel is metal and is commonly used in stainless steel production. Stainless steel that includes nickel is used in the food processing, transportation, and manufacturing industries because of its advantages such as being heat-resistant, resistant to damage, and easy to keep clean. It is commonly used in everyday household items as well [19].

**Figure 1:**
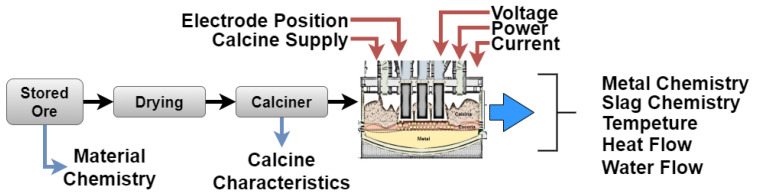
Steps in the Ferronickel extraction process.

**Figure 2:**
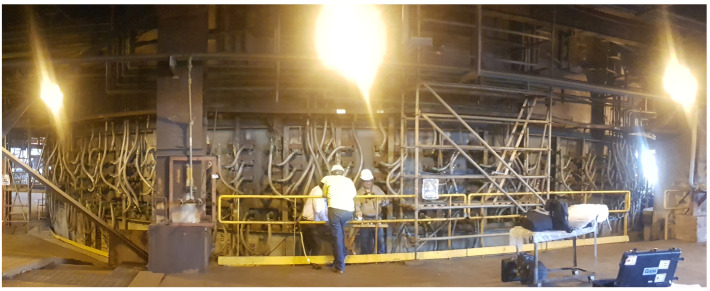
Panoramic of the furnace.

In the case of CMSA, the main product is ferronickel (FeNi), which is a material used in different industries such as electronics, manufacturing, and automotive, among others. This material is obtaining in a metallurgical procedure after mining laterites and high-Ni sulfide ores [20]. Figure 1 shows a diagram depicting the ferronickel extraction process. This process starts with the ore extraction in an open-cut mine, which is tested and classified. The stored ore is dried in a calcine furnace (kiln furnace) and afterward processed in an electric arc furnace where the smelting process is performed. Figure 2 shows a panoramic of a section of the furnace. Although all parts of the process are important, the monitoring of the furnace is a vital task because of the risks associated with its operation. It is necessary to remark that this element works 24/7, and the aim of the monitoring system is to reduce the number of maintenance activities by an enhanced operation of the furnace. The experience of the furnace operators, who are making decisions based on the data from the process every day, is one of the main sources of knowledge that can make this happen, but for they this they require good online information and predictions.

### 2.3. Deep Learning Predictive Methods

As is shown in Figure 1, the ferronickel extraction process requires several steps and involves multiple inputs and outputs. This complexity requires the use of advanced strategies for data analysis and it is here where neural networks present solutions to tackle problems that involve sequential processing of data [21]. The goal of this paper is to develop a predictor of the temperature behavior in the furnace using mainly data obtained from thermocouple sensors located at the lining furnace. One of the requirements of the proposed method is to effectively model long-term dependencies between variables, therefore it is necessary to use information from earlier time windows (past information) in order to be able to accurately predict the temperature (future predictions). This highlights the fact that it is necessary to develop models that can handle variable-length input sequences, that are able to track long-term dependencies in the data, that can maintain information about the order of the sequences, and share parameters across the entirety of the sequence.

Feedforward neural networks are not able to maintain information about a previous event in a sequence of events [22]. In contrast, recurrent neural networks (RNN) have loops in their architecture, which allows for information to persist over time. These networks are called recurrent because the information is being passed from one time-step to the next internally within the network [23].

The RNNs use a training algorithm called backpropagation through time [24]. Errors are backpropagated at each time step, and then, finally across all time steps all the way from where we are currently to the beginning of the sequence [25]. This is the reason why it is called backpropagation through time. The computation of the gradient, that is the derivative of the loss with respect to the parameters tracing all the way back to the initial state, requires many repeated multiplications of the weight matrix as well as repeated use of the derivative of the activation function. In some cases, this is a problem because gradients are too small. This problem is well-known as the vanishing gradient problem [26]. There are three different ways to overtake the vanishing gradient problem: (i) choosing the activation function, (ii) initializing the weights cleverly (close to the optimal solution), and (iii) designing the network architecture to actually be able to handle this efficiently. This work focuses on the use of the latter solution, which uses a slightly more complex recurrent unit that can track long-term dependencies in the data more effectively by controlling what information is passed through and what information is used to update its internal state, i.e., the gated cell. Two types of gated cells are described next, namely the long short-term memory (LSTM) and the gated recurrent unit (GRU).

#### 2.3.1. Convolutional Neural Networks

Convolutional neural networks (CNNs) are feedforward artificial neural networks that use the convolution operation instead of matrix multiplication. The main aspects of the convolution layer in CNNs are its sparse local connectivity and filters, which significantly reduce the number of network parameters, while simultaneously increasing its performance [27]. Features in CNN are not hand engineered, but learned; this property reduces the preprocessing stage.

#### 2.3.2. Long Short-Term Memory Network

**Figure 3:**
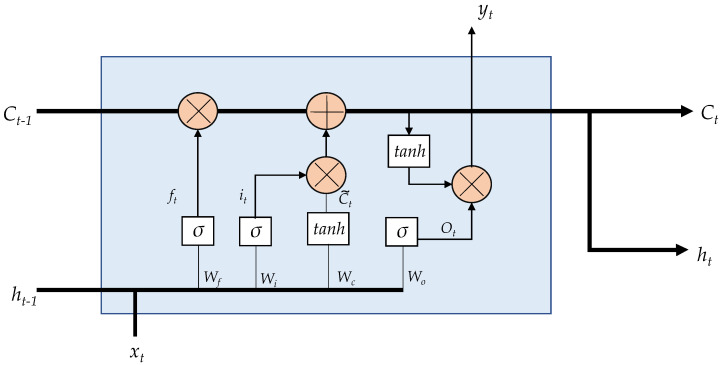
LSTM internal unit configuration.

In an LSTM network, the repeating unit contains different interacting layers. These layers interact to selectively control the flow of information within the cell. This enables LSTM to track and store information throughout many time steps. The key building block behind the LSTM is the gate, which functions to enable the LSTM to selectively add or remove information to its cell state. LSTM processes information through four simple steps: forget, store, update, and output. These networks must first forget irrelevant history, then perform the computation to store relevant parts of new information, use these two steps together to selectively update their internal state, and finally, generate an output. The internal description of an LSTM unit is illustrated in Figure 3. LSTM considers an input sequence $\left\{x_{1},\;x_{2},\ldots ,\;x_{t}\right\}$ at time *t*. The output gate determines the new state $h_{t}$, where *t* is the time step. The following equations describe the internal operations carried out in an LSTM internal unit [28].

(1) $$f_{t}=\sigma \left(W_{f}\times \left[h_{t-1},x_{t}\right]+b_{f}\right)$$

(2) $$i_{t}=\sigma \left(W_{i}\times \left[h_{t-1},x_{t}\right]+b_{i}\right)$$

(3) $$o_{t}=\sigma \left(W_{o}\times \left[h_{t-1},x_{t}\right]+b_{o}\right)$$

(4) $${\tilde{C}}_{t}=tanh\left(W_{c}\times \left[h_{t-1},x_{t}\right]+b_{c}\right)$$

(5) $$C_{t}=f_{t}\times C_{t-1}+i_{t}\times {\tilde{C}}_{t}$$

(6) $$h_{t}=o_{t}\times \tanh\left(C_{t}\right)$$

where, $C_{t-1}$, ${\tilde{C}}_{t}$, and $C_{t}$ are unit memory, $W_{c}$, $W_{o}$, $W_{f}$, and $W_{i}$ are weight matrices; $b_{c}$, $b_{o}$, $b_{f}$ and $b_{i}$ are bias vectors.

#### 2.3.3. Gated Recurrent Unit

The GRU [29] is a modified version of the LSTM cell. It combines long and short-term memory into its hidden state. The GRU has two gates, on one hand, the *update* gate and on the other hand the *reset* gate. These gates allow maintaining a balance between the information to retain and forget. The outputs of the GRU unit are $z_{t}$ (see Equation (7)) and $r_{t}$ (see Equation (8)).

**Figure 4:**
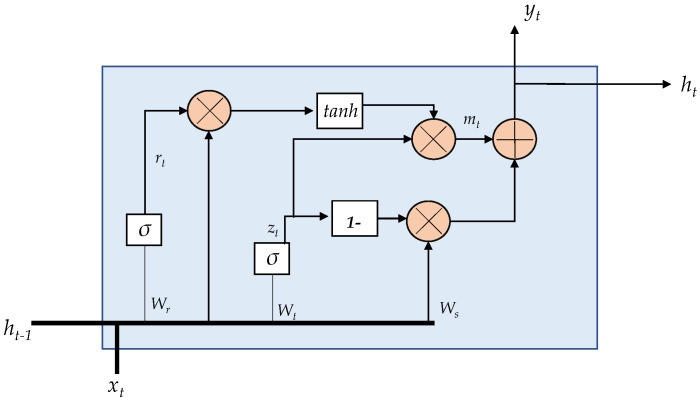
GRU internal unit configuration.

The internal description of a GRU unit is depicted in Figure 4. The following equations describe the internal operations in the GRU unit [28].

(7) $$z_{t}=\sigma \left(W_{z}\times \left[h_{t-1},x_{t}\right]+b_{z}\right)$$

(8) $$r_{t}=\sigma \left(W_{r}\times \left[h_{t-1},x_{t}\right]+b_{r}\right)$$

(9) $$m_{t}=\tanh\left(W_{s}\times \left[r_{t-1},x_{t}\right]+b_{s}\right)$$

(10) $$h_{t}=\left(1-z_{t}\right)\times h_{t-1}+z_{t}+m_{t}$$

where, $W_{r}$, $W_{z}$ and $W_{s}$ are weight matrices; and $b_{r}$, $b_{z}$, and $b_{s}$ are bias vectors.

## 3. Temperature Prediction Methodology for the Furnace Lining

**Figure 5:**
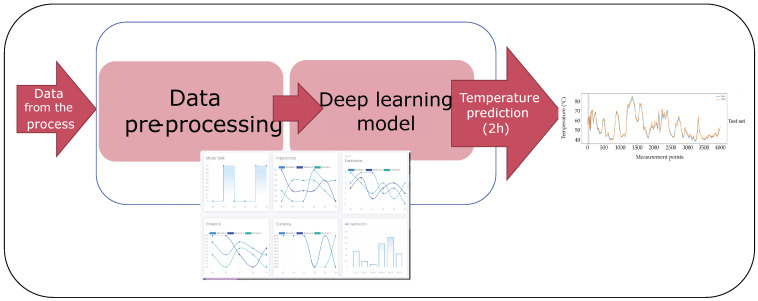
General steps in the temperature prediction methodology.

This section is devoted to introducing the proposed methodology for temperature prediction in the wall of the ferronickel furnace studied. Two general steps are considered after the data acquisition step: the data cleaning process and the development of the deep learning model as depicted in Figure 5. Although the methodology considers some particular elements and variables of this specific smelting process, it can be generalized to other complex processes where a big number of sensors are used and it is necessary to predict the behavior of a variable.

Before presenting each step of the methodology, some context with regards to the data set obtained from the data acquisition system is given next.

### 3.1. Dataset for Methodology Validation

The data set corresponds to the measured variables of an electric arc furnace for ferronickel production located at Cerro Matoso SA (South32 company). The data set used was sampled every 15 min for a period of 416 days between the 11 August 2018 and 30 September 2019. The data set is composed of a total of 40,000 instances. Regarding the attributes, also called variables or features, an in-depth analysis with a group of expert furnace operators defined a group of 49 variables selected due to their importance in the furnace operation. These 49 variables are detailed in Table 1 and serve as input variables to train and test the developed multivariate time series temperature prediction system.

**Figure 6:**
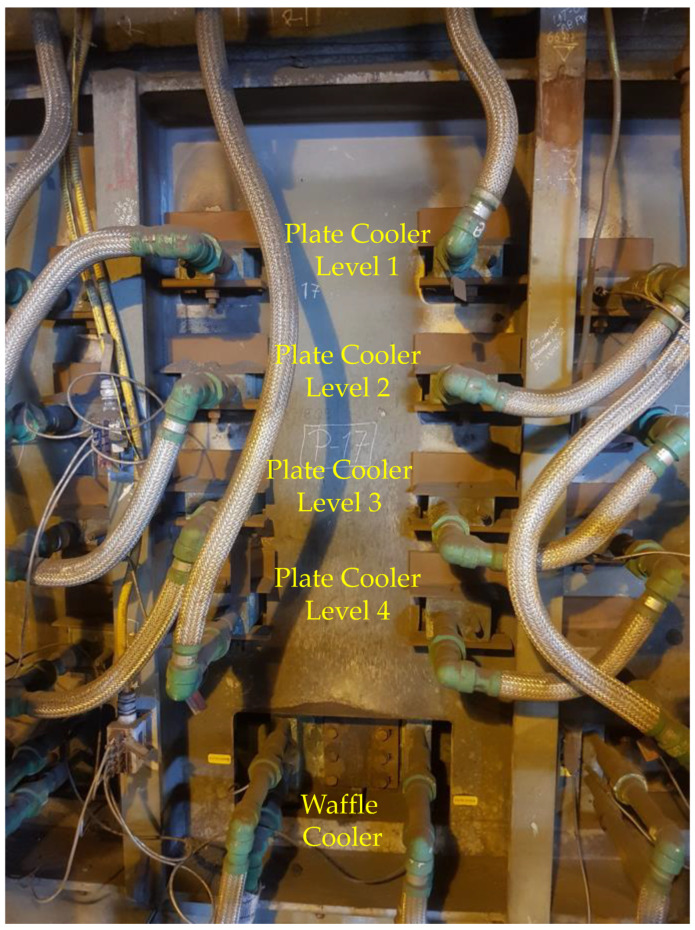
External view of panel #17 of the 72 panels wallside that radially compose the furnace.

The electric arc furnace is built with the integration of 72 panels radially distributed on its perimeter. Each panel is composed of four plate coolers; these four plate coolers are located at four different heights labeled Level 1, Level 2, Level 3, and Level 4 as depicted in Figure 6. Thus, there are 72 × 4 = 288 plate coolers radially distributed along the furnace. Each plate cooler has a thermocouple for temperature lining monitoring. Figure 6 shows a section of the wall of the furnace where it is possible to observe the refrigeration system composed of 4 levels of plate coolers per panel, where the thermocouples, whose temperature measurement must be predicted, are located.

**Figure 7:**
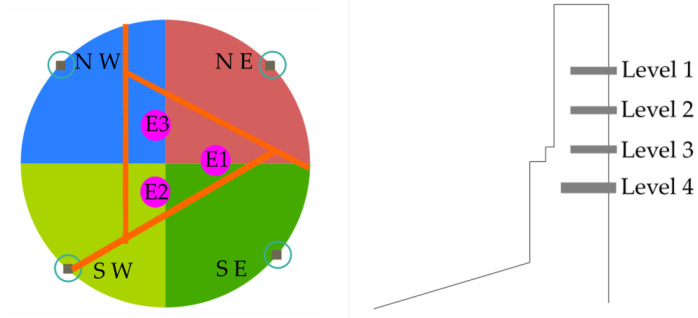
(**Left**) radial location of the 4 panels to evaluate and (**Right**) detail side view of each panel with its 4 levels of plate coolers. Sixteen thermocouples in total were used as output variables.

Due to the high number of plate cooler thermocouples in the lining furnace, a discrete group of 16 thermocouples were selected in this work as output variables to be predicted. Four panels of the furnace belonging to the North-West (NW), South-West (SW), South-East (SE), and North-East (NE) quadrants were selected. Each panel has four thermocouples, thus a 4 × 4 = 16 thermocouples in total were selected. The distribution of these 16 thermocouples is illustrated in Figure 7. The location of the three furnace electrodes (E1, E2, and E3) is detailed in Figure 7 (left).

### 3.2. Data Pre-Processing Step

**Figure 8:**
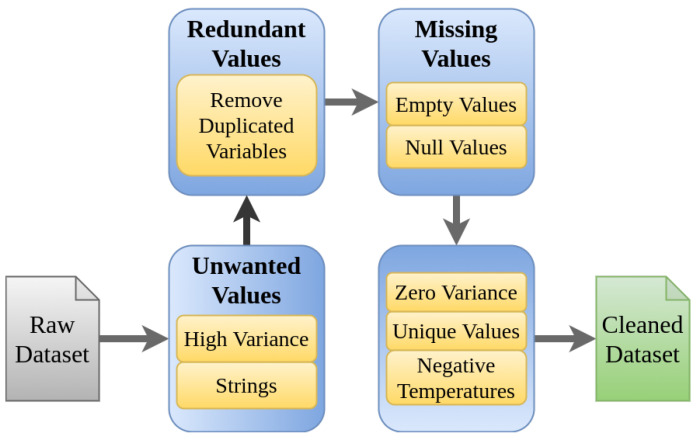
Data cleansing process workflow.

The ferronickel production process carried out at the CMSA facilities is made up of a large number of variables. Variables are acquired from different sources, including multiple on-line sensors, and collected by a data acquisition system (DAQ). The collected data can contain errors because of failures in sensors, noise, or missed data that the system fails to capture or store. These errors require revision and errors must be eliminated in the pre-processing step in order to reduce the errors in the model to be developed. Originally 1180 variables were provided by CMSA. Together with experts from the process operations area, some considerations about the range of the variables were identified, and the elements shown in Figure 8 were defined as the set of rules to be considered. These elements constitute a workflow with seven steps, which contains different types of problems that might be present in the initial data set, for example, strings of characters in numeric variables, negative temperatures, variables that remain in a single value, variables with null data. With this workflow, it was possible to find variables that consistently presented problems in the data, and for this reason, they were eliminated. It is also noted that given the large number of variables available, it was not necessary to carry out the data restoration processes, avoiding in this way the appearance of gaps in the data set. The results obtained after applying the proposed workflow allowed to debug 340 variables with errors, which indicates that 28% of the data has problems.

**Figure 9:**
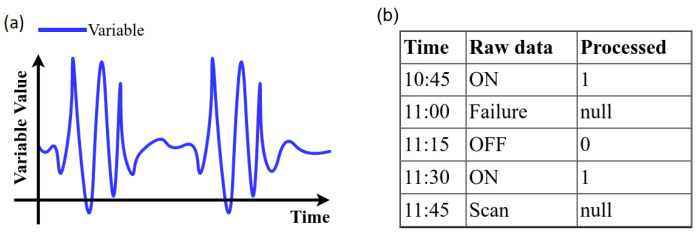
Data cleansing process rules: (**a**) variables with high variance and (**b**) strings.

**Figure 10:**
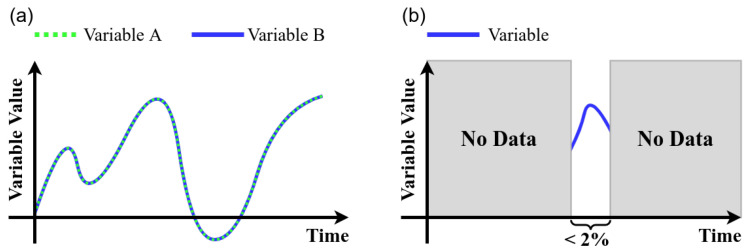
Data cleansing process rules: (**a**) *remove duplicates* and (**b**) *empty and null values*.

**Figure 11:**
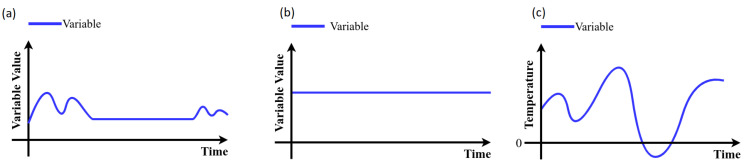
Data cleansing process rules: (**a**) variables which remain in same value for long time periods, (**b**) unique values, and (**c**) negative temperature cases.

The following steps in the data cleaning process were considered [15]:

- *High variance*: Data with values outside of the operational range should not be considered. Thus, a univariate measure for the measurement of quality based on percentage changes is calculated. A variable representation with variance is shown in Figure 9a.
- *Strings*: the variables that had non-numeric values were encoded with numerical values (see Figure 9b).
- *Remove duplicates*: The identification of duplicate variables results in drop the duplicate and stay with a single variable (see Figure 10a).
- *Empty and null values*: A 2% was selected as threshold, thus, variables with more than 98% with empty or null values are dropped. (see Figure 10b)
- *Zero variance*: If more than 50 % of the data of a variable does not vary and remains in a constant value the variable is dropped (Figure 11a).
- *Unique values*: Because it is desirable to find relationships between variables those that remain constant over time are dropped (see Figure 11b).
- *Negative temperatures*: The normal operating range of variables that measure temperature always takes positive values. Due to the above, some variables with negative temperatures are identified and these are dropped (Figure 11c).

The amount of variables eliminated in each of the categories in the data cleaning process is described in Table 2. As a result, after performing the cleaning process, 840 variables were obtained in a cleaned data set. Subsequently, through suggestions made by the furnace operators and the judgment of expert engineers belonging to CMSA, the 49 variables of the temperature prediction model developed in this work were selected. These 49 variables are listed in Table 1.

### 3.3. Development of the Multivariate Time Series Deep Learning Model

The following section will the steps carried out in order to achieve our goal of training a temperature prediction model. To this end, we will first discuss the steps taken in order to define a suitable data set. This is followed by a discussion on the definition and development of the RNN proposed to be used in this paper.

The programming language selected to implement the temperature prediction models is Python. Together with this programming language the following libraries are used for data management, neural network training and visualization, among other functions:

- Data manipulation: Pandas and NumPy
- Data visualization: Matplotlib and Seaborn
- Neural networks training: scikit-learn [30], TensorFlow [31], and Keras [32].

#### 3.3.1. Definition of the Dataset

The data set has input variables of different magnitudes and values. So the different values are scaled so that they are in the interval between −1 and 1. The target data comes from the same data set as the input signals, because they are the output thermocouple data that simply shifts in time.

The number of time steps that it will shift the target data is predefined. The dataset was sampled to have one observation every 15 min, thus there are 96 observations over 24 h. In particular, the shift is used to predict temperatures two hours in the future.

Due to the large number of instances that were taken in the dataset (40,000) it is impractical to perform a conventional division of 70% of the data for training and the remaining 30% for testing. In contrast, and due to the desire to have the greatest amount of data for training, the decision was made to divide the data into 90% for training and the remaining 10% for testing. In addition, the input and output variables for the training and test sets were defined. The dataset must be prepared as two-dimensional NumPy arrays. In this case, there are 49 input signals and 16 output signals.

Instead of training the recurrent neural network on the entire sequence of 40,000 observations, a function is used to create a batch of shorter 250 subsequences randomly selected from the training data. Thus, every sequence had a size of =1152 steps corresponding to 12 days. This period of time is defined because it is in which the pile of calcined material in the furnace is changed. The 40,000 data used in the training and testing of the temperature prediction model were cleaned before the development of the neural network. Thus, there are 250 random sequences (batch) with a size of 1152 steps and all the data belonging to this batch is clean following the steps described in Section 3.2, where it was determined as a data cleaning policy that if a variable had any of the problems indicated, it would be eliminated, this was possible given a large amount of information available and variables in the initial data set. For that reason, the variables with data problems were not available for the training data set, because their information did not represent the real behavior of the furnace, therefore it is clarified that there was no partial elimination of values that generated discontinuity in the time series.

As described, the training is performed by taking 250 random sequences. However, to carry out the testing, the complete sequence of data is taken in the test set corresponding to 4000 records. These 4000 records in the test set are sufficient and still providing a good enough estimate of the model performance. In addition, the model performance is monitored after each epoch on the test set and only if the performance is improved on the test set the weights of the recurrent neural network are saved for the next epoch.

#### 3.3.2. Creation of the Recurrent Neural Network

The neural network and its different layers are created using TensorFlow in a sequential model. The first layer consists of the use of a cell type gated recurrent unit (GRU) to create a recurrent neural network. This GRU layer had 250 outputs for each time step in the sequence. The information that enters this first GRU layer of the model is a batch of sequences of arbitrary length where each observation has several input signals. The GRU network generates a batch of sequences of 250 values. You want to predict 16 output signals; thus, a dense layer is added in the deep learning model that assigns 250 values to only 16 output values corresponding to the 16 thermocouples that it is desired to predict. A sigmoid activation function is used to ensure that the values are within the normalized values.

After each epoch of the neural network, the performance of the model in the test set is monitored and the model weights are only saved if the performance improves in the test set. In the training process, a batch of short sequences is randomly selected from the total training data. In this case the training data has 36,000 instances. In contrast, for the validation data, the entire sequence is run from the 4000 instances in the test set and the prediction accuracy is measured on that entire sequence.

**Figure 12:**
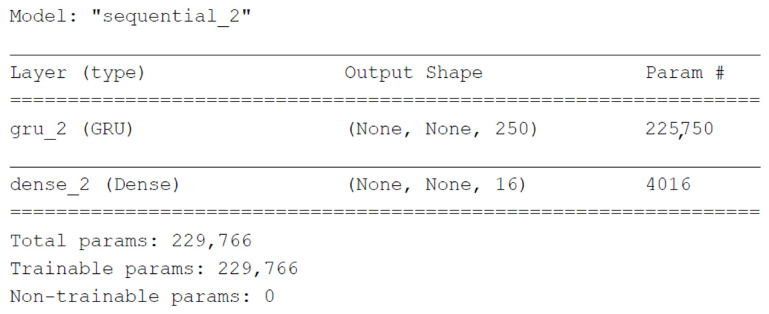
Output shape in the GRU + Dense model.

It is important to discuss the loss function, learning rate, and warmup period. The loss function that is minimized is the mean square error (MSE). A warmup period of 50 time steps is assigned to the model so that the precision of these first 50 steps is not used in the stall function. The inclusion of this warmup period allows the model to present a better behavior for each of the 16 outputs in terms of root mean square error RMSE. Adam [33] was selected as an optimizer and an initial learning rate of $1\times 10^{-3}$ is used. If the loss of validation has not improved since the last epoch, the learning rate changes to $1\times 10^{-4}$. A two-layer model was defined, one GRU and the other dense. The output form of (None, None, 16) shown in Figure 12 means that the model will generate a batch with an arbitrary number of sequences, each of which has an arbitrary number of observations, and each observation has 16 output signals.

#### 3.3.3. Training of the Recurrent Neural Network

A single “epoch” does not correspond to a single training set processing, due to how the batch generator randomly selects subsequences from the training set. Instead, “steps-per-epoch” is selected to have an epoch processed in a few minutes. In this case, the number of steps-per-epoch used is equal to 100. The parameters of the joint GRU + Dense model are described in Table 3.

#### 3.3.4. Performance of the On-Line Prediction Model

The final step is to compare the predicted and true output signals. The time series prediction model performance is calculated using the root mean squared error (RMSE):

(11) $$RMSE=\sqrt{\frac{1}{M}\sum_{i=1}^{M}{\left({\tilde{y}}_{i}-y_{i}\right)}^{2}}$$

where *M* is the number of data points in the time series to be estimated, $y_{i}$ is the actual value of time series, and ${\tilde{y}}_{i}$ is the estimated value at time *i* by the prediction model [34].

**Figure 13:**
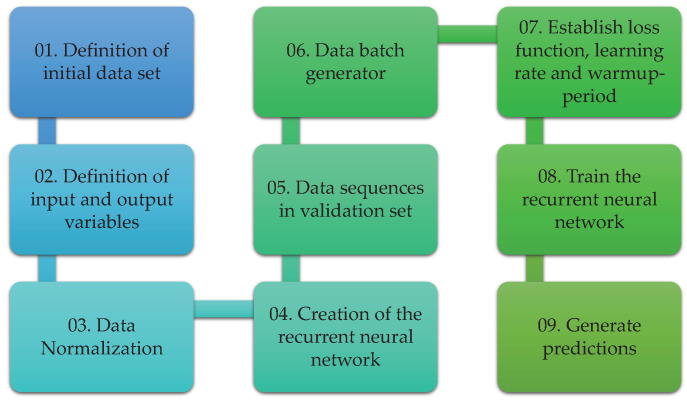
Steps for the development of the temperature prediction deep learning models.

The steps to develop the different deep learning models to predict the temperature are described in this section. These steps are illustrated in Figure 13.

## 4. Results and Discussion

**Figure 14:**
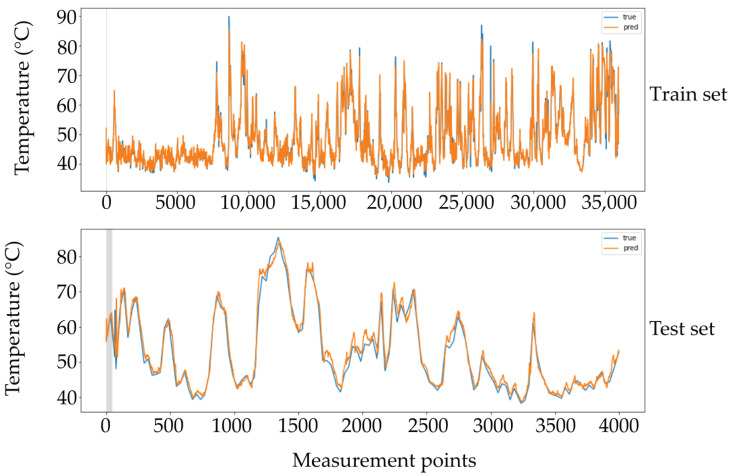
True versus predictive behavior of the gated recurrent unit (GRU) model. Training and test sets in one of the output thermocouples.

As a result of the multivariate time series deep learning model evaluation, a comparison of the predicted and true behaviors for one thermocouple in the train and test sets is shown in Figure 14. It can be deduced the similarity between the true and predicted values in both cases.

### 4.1. RMSE Study in the Train and Test Sets for Six Different Deep Learning Models

**Figure 15:**
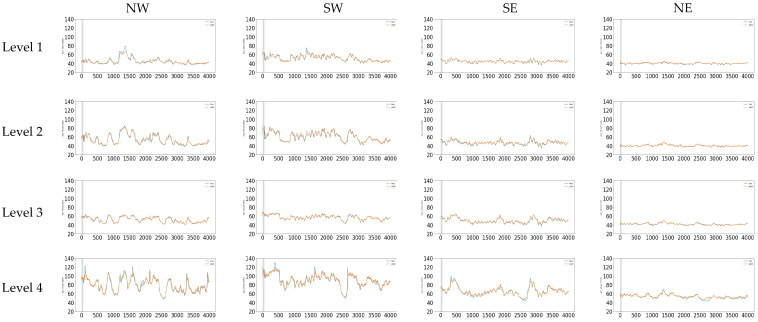
True and predicted behavior in each one of the 16 thermocouples studied in the test set.

A comparative study showing the 16 thermocouple results in the test set is shown in Figure 15. In each subfigure, the true value is depicted in blue and the predicted value is depicted in orange. From Figure 15 is evident that Level 1 reaches the lowest temperatures in general independently of the quadrant. In contrast, the highest temperatures in the furnace were reached in Level 4 due to its bottom location in the furnace. When a comparison between quadrants is performed, it is evident that the NW and SW sectors exhibit higher values than SE and NE sectors in the furnace. This is probably due to an erosion in the refractory lining in the NW and SW quadrants of the furnace.

Table 4 shows the RMSE values for each one of the 16 thermocouples for the train and test sets. A comparison study of the RMSE behavior was carried out with six different deep learning models. These deep learning models were:

- GRU (250 Cells) + Dense
- GRU (128 cells) + GRU (64 cells) + Dense
- LSTM (250 cells) + Dense
- GRU (128 cells) + LSTM (128 cells) + Dense
- CONV1D (128 cells) + Dense
- CONV1D (128 cells) + LSTM (128 cells) + Dense

As shown in Table 3 the best deep learning model was the GRU (250 Cells) + Dense. It may be observed that the best average RMSE value of 1.19 in the test set was obtained by the GRU (250 Cells) + Dense model. The CONV1D layer automatically extracts features from the input time series during training. As can be observed in Table 3 the second-best model was the CONV1D (128 cells) + Dense reaching an average RMSE value of 1.48 in the test set. This behavior was obtained by using one-dimensional filters to capture the temporal properties in the CONV1D layer to describe the temporal pattern of the input series [35]. The thermocouples in Level 1 NE and Level 2 NE reached the lowest RMSE values. In this case, the two aforementioned thermocouples belong to the NE plate cooler and correspond to the upper and middle Levels 1 and 2 showing their high correlation. In general, the NE quadrant exhibits the best RMSE values and it is evident that the NE quadrant presents the lower temperatures of the entire furnace.

Particularly, the thermocouple in Level 4 NE reached a maximum temperature of 70 °C. In contrast, the thermocouples in Level 4 NW and SW reached temperatures above 120 °C. The worst RMSE values were obtained for the thermocouples of Level 4 NW and Level 4 SW, with values of 2.94 and 2.61 respectively in the test set. This behavior is mainly due to the influence of the electrodes at this position in the furnace area.

**Figure 16:**
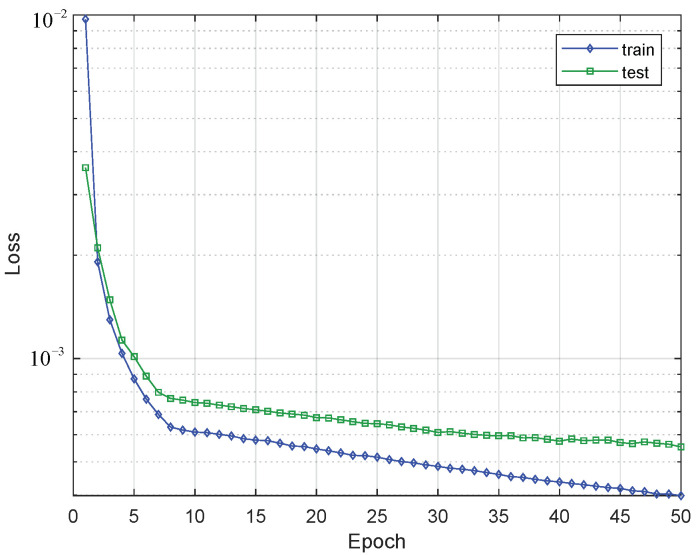
Changing of the loss value through the epocs in train and test sets of the GRU (250 Cells) + Dense model.

The loss behavior of the training and test sets is shown in Figure 16. A change in behavior is evident when the model reaches epoch 8 since, from then on, the decrease in the loss presents less intensity. Due to the small values of the loss in magnitude a logarithmic y-axis is used. It is evident that the values of the training set are lower than those of the test set as the epochs increase. Additionally, the observed behavior allows us to obtain a model while preventing over-fitting.

### 4.2. Robustness Study against the Random Sampling in the Sequences in the Training Set

Table 5 shows the average RMSE behavior for train and test sets after performing five iterations for the GRU (250 Cells) + Dense model. All the parameters remained constant in every iteration. The slight changes are due to the random sampling in selecting 100 sequences to train. From Table 5 it is evident that the RMSE remains in the same order of values through the iterations. Therefore, the robustness of the model can be evidenced.

### 4.3. Study of the Variation of the Number of Sequences in the GRU Model

A next study changing the size of sequences that are part of the batch in the training is performed to evaluate the RMSE behavior of the GRU (250 Cells) + Dense model. The variation results of the size of sequences using 10, 50, 100, 150, 200, and 250 random sequences in the training is depicted in Table 6. The best average RMSE value in the test set was achieved when using 250 random sequences.

### 4.4. Moving Origin Four-Fold Cross Validation Strategy in a Time Series Approach

**Figure 17:**
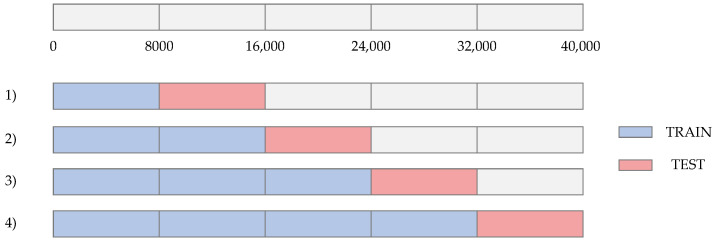
Four-fold moving origin cross validation strategy in the time series approach.

A four-fold moving origin [36] cross-validation strategy in a time series approach was performed. The moving origin four-fold cross-validation strategy is depicted in Figure 17. This cross-validation strategy is carried out by changing the percentage of data in the train and test sets in different iterations. Due to the total number of instances is 40,000 a four-fold split was selected. As a result, four different folds were evaluated. The size of the test set in each fold is equal to 8000 data. The results of the average RMSE values obtained in the train and test sets in each fold are shown in Table 7. It is evident the low RMSE value of 0.52 in the train set for fold #1. As the iterations are performed and the amount of data in the train set increases, the average RMSE also increases in the train set. In contrast, a decreasing average RMSE behavior across the folds is evident in the test set. This is a consequence of the increased data in the train set, which allows for better modeling of the furnace behavior.

### 4.5. Sensitiveness against Training/Test Ratio

A study of the sensitiveness against training/test ratios of 90/10, 85/15 and 80/20 was performed on the entire 40,000 data. The results of average RMSE are shown in Table 8. An average RMSE value remained constant for the train set in all the cases. In contrast, the behavior of the average RMSE increased in the test set as the size of the test set increased from 10% to 20%. The best average RMSE of 1.19 for the test set was obtained for the 10% configuration, this indicates that the more data that belongs to the training set, the better the model will be able to generalize the behavior of the 16 output thermocouples.

### 4.6. Influence of the Variables Used in the Model

**Figure 18:**
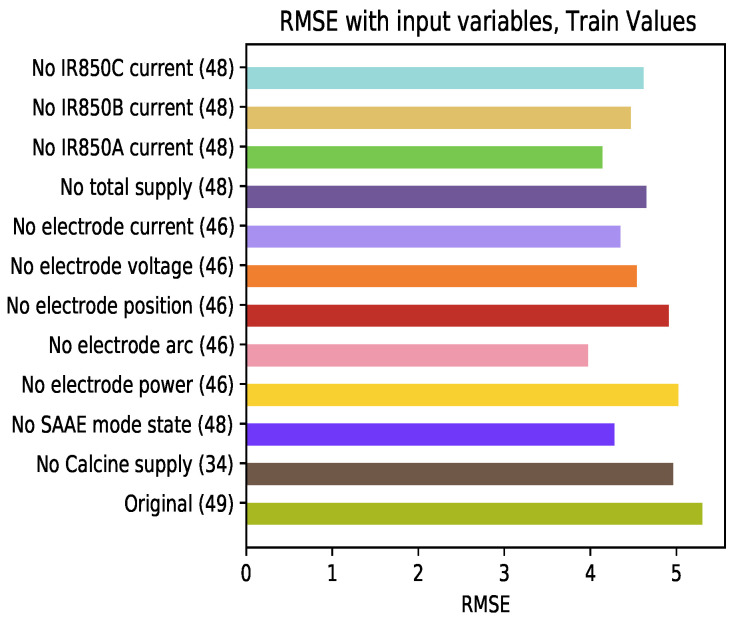
Average RMSE of the train set varying the learning variables at the input of the model.

To determine the behavior of the model in relation to the input variables, the GRU model was trained by eliminating one variable at a time. Figure 18 shows the results of RMSE for this analysis in the training set. A low influence of some of the modifications is observed, for example, the omission of inputs related to the furnace power. On the other hand, the variables related to the electrodes arc have a considerable influence on the decrease of errors in training.

**Figure 19:**
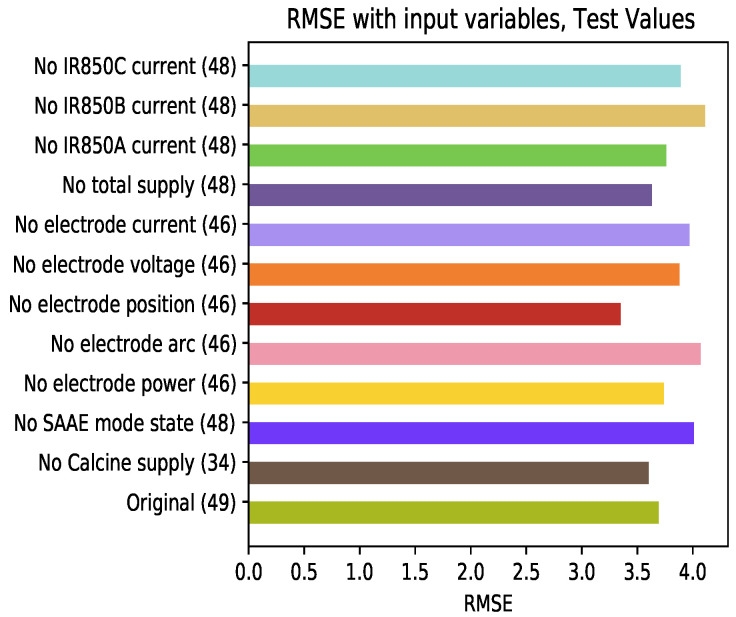
Average RMSE of the test set varying the learning variables at the input of the model.

Figure 19 shows the results of the model’s test set. Remarkable changes are observed in relation to the test results. The elimination of the variables related to the position of the electrodes (being the variable that decreases the RMSE the most) is the omission that has the greatest influence on the increase in this error. The complexity of the developed prediction model can be simplified by reducing the number of inputs, specifically those related to the position of the electrodes, without affecting the results in the training and testing processes. For example, it can be observed that the variables electrode position and calcine supply are not improving the model performance. However, it can be observed that current variables are affecting the RMSE when they are removed, hence they are important to the model.

## 5. Conclusions

In this paper, a multivariate time series deep learning model was developed to predict the temperature behavior in an electric arc furnace. The developed temperature prediction methodology was tested on a dataset of 416 days of an electric arc furnace operation, corresponding to 40,000 instances. Sixteen thermocouples radially distributed in the furnace at four different height levels were selected as output variables. The results yielded by the GRU (250 Cells) + Dense deep learning model showed an average RMSE of 1.19 °C for the test set using a training/test ratio of 90/10. This shows the goodness of the prediction in the SHM system for furnace lining temperature monitoring.

It was found that approximately 28% of the original dataset presented abnormalities, thus, it was very important to carry out a data preprocessing step including data cleansing, outlier removal, and removing redundant, null, and unwanted values.

The developed deep learning model allowed us to perform temperature predictions in the lining of the furnace at 2 h in the future. Consequently, the predicted behavior of the furnace facilitates decision-making associated with the possible high temperatures of the furnace hearth due to changes in the operational variables. These predictions contribute to carrying out correct structural health monitoring and preventive control of the furnace lining erosion, caused by excess temperature.

This research allowed us to determine which variables are relevant in the prediction of temperature, confirming the hypothesis about the relationship between each variable and the furnace lining temperature. This was necessary to determine the input variables of the multivariate time series deep learning model.

In future work, an attention-based model inspired by an encoder-decoder approach will be applied to predict the furnace temperature considering relations between variables in long and short term periods of time. Moreover, a more sophisticated architecture for the CNN1D, involving several layers of convolution and maxpooling will be proved in order to identify their capacity to catch more abstract features. Besides, the developed model will be tested in another electric arc furnace, and their ability to predict its lining temperature will be compared.

## Figures and Tables

**Table 1:** Description of input variables.

Variable Identification	Number of Input Variables
calcine feed totalizer	1
automatic furnace regulation system	1
electrode current	3
electrode voltage	3
relative electrode position	3
electrode arc	3
electric furnace power	1
electrode power	3
chemical composition of calcine	15
16 thermocouples to predict	16
Total input variables	49

**Table 2:** Amount of variables eliminated in each of the categories after performing the data cleaning process.

Category	Deleted Variables
High variance	97
Strings	5
Remove duplicates	80
Empty and null values	2
Zero variance	22
Unique values	60
Negative temperatures	74
Total deleted variables	340

**Table 3:** Parameters setup in the GRU + Dense model.

Parameter	Value
Neural network layer type	GRU + Dense
Number of GRU grid cells	250
Optimization method	adam
Learning rate	$1\times 10^{-3}$
Number of training epochs	50
Steps per epoch	100
Total number of data	40000
Data division percentage for training	90%
Data division percentage for testing	10%
Number of random sequences belonging to the batch for training	250
Number of steps in each random sequence	1152
Number of shift steps	8 every 15 min, predicts 2 h into the future
Number of input variables	49
Number of output variables	16

**Table 4:** RMSE comparison of six different deep learning models for each one of the 16 output thermocouples in the training and test sets. (The percentage of data split in train set was 90% and the remaining 10% was used for test set.)

	GRU 250		GRU 128 + GRU 64		LSTM 250		GRU 128 + LSTM 128		CONV1D 128		CONV1D 128 + LSTM 128	
**Thermocouple**	**Train**	**Test**	**Train**	**Test**	**Train**	**Test**	**Train**	**Test**	**Train**	**Test**	**Train**	**Test**
Level 1 NW	0.66	1.34	1.03	1.86	0.80	1.81	0.95	1.82	0.88	1.64	0.87	1.74
Level 1 SW	0.87	1.10	1.46	1.83	1.09	1.77	1.27	1.89	1.11	1.24	1.15	1.56
Level 1 SE	0.65	0.71	0.89	0.85	0.76	1.15	0.87	1.02	0.78	0.73	0.72	0.82
Level 1 NE	0.45	0.43	0.75	0.60	0.58	0.61	0.64	0.59	0.60	0.53	0.60	0.52
Level 2 NW	1.08	1.46	1.58	1.80	1.31	2.05	1.47	2.46	1.54	1.99	1.38	2.03
Level 2 SW	1.09	1.54	1.88	2.41	1.41	2.19	1.62	2.70	1.58	1.68	1.45	2.26
Level 2 SE	0.73	0.89	1.10	1.32	0.84	1.41	0.98	1.43	0.94	1.18	0.83	1.12
Level 2 NE	0.50	0.43	0.74	0.59	0.65	0.73	0.71	0.69	0.64	0.54	0.61	0.74
Level 3 NW	0.81	0.85	1.13	1.05	0.95	1.21	1.03	1.24	1.00	1.07	0.98	1.42
Level 3 SW	0.80	0.77	1.28	1.16	1.01	1.20	1.17	1.33	1.02	1.04	1.06	1.10
Level 3 SE	0.76	0.96	1.09	1.21	0.88	1.47	0.98	1.48	0.91	1.01	0.80	1.05
Level 3 NE	0.54	0.47	0.76	0.62	0.62	0.74	0.69	0.66	0.66	0.70	0.62	0.55
Level 4 NW	2.40	2.94	3.28	3.83	2.86	3.94	3.03	4.46	2.99	3.53	2.65	3.08
Level 4 SW	2.28	2.61	3.35	3.49	2.77	3.88	3.10	3.63	3.02	3.11	2.62	3.38
Level 4 SE	1.67	1.59	2.13	2.09	1.94	2.33	2.01	2.26	1.74	1.85	1.80	1.93
Level 4 NE	0.94	1.02	1.41	1.71	1.14	1.84	1.36	1.89	1.29	1.86	1.16	1.59
**Average**	**1.01**	**1.19**	1.49	1.65	1.23	1.77	1.37	1.85	1.29	1.48	1.21	1.56

**Table 5:** Average RMSE behavior in training and test sets in five different iterations for the GRU (250 Cells) + Dense model. The number of sequences in batch train was set to 100 in all cases.

	Iteration 1	Iteration 2	Iteration 3	Iteration 4	Iteration 5
Train	1.07	1.07	1.1	1.04	1.1
Test	1.24	1.26	1.26	1.23	1.26

**Table 6:** Average RMSE behavior in train and test sets changing the number of random sequences for training the GRU (250 Cells) + Dense model.

	Number of Random Sequences for Training					
	**10**	**50**	**100**	**150**	**200**	**250**
Train	1.08	1.06	1.1	1.05	1.06	1.01
Test	1.26	1.22	1.26	1.3	1.29	1.19

**Table 7:** Average RMSE moving origin four-fold cross validation results in the training and test sets for the GRU (250 Cells) + Dense model.

	Fold 1	Fold 2	Fold 3	Fold 4
Train	0.52	0.7	0.95	1.02
Test	4.93	2.31	1.64	1.60

**Table 8:** Average RMSE sensitiveness analysis of three different training/test ratios 90/10, 85/15 and 80/20.

	90/10	85/15	80/20
Train	1.01	1.00	1.02
Test	1.19	1.54	1.60

## Abbreviations

The following abbreviations are used in this manuscript:

LSTM	Long Short Term Memory
GRU	Gated Recurrent Unit
CNN	Convolutional Neural Network
RNN	Recurrent Neural Network
DCS	Distributed Control System
